# Traversing the data landscape: insights and recommendations from a case study using novel linkage of care home and health data

**DOI:** 10.1136/bmjhci-2025-101600

**Published:** 2026-01-12

**Authors:** Elizabeth Crellin, Kaat De Corte, Freya Tracey, Jennifer Kirsty Burton, Stacey Rand, Stephen Allan, Arne Timon Wolters, Claire Goodman, Therese Lloyd

**Affiliations:** 1 The Health Foundation, London, UK; 2 Academic Geriatric Medicine, School of Cardiovascular and Metabolic Health, College of Medical, Veterinary and Life Sciences, University of Glasgow, Glasgow, UK; 3 Care and Outcomes Research Centre (COReC), University of Kent, Canterbury, UK; 4 Northumberland Tyne and Wear NHS Foundation Trust, Newcastle upon Tyne, UK; 5 NIHR Applied Research Collaboration North East and North Cumbria, Gosforth, UK; 6 Centre for Research in Public Health and Community Care, University of Hertfordshire, Hatfield, UK; 7 NIHR Applied Research Collaboration East of England (NIHR ARC EoE), Cambridge, UK

**Keywords:** Medical Record Linkage, Health Services Research, Data Management

## Abstract

The insights available from linking routine health data have transformative potential for understanding and improving population health and well-being. However, cross-sectoral data linkage in the UK remains challenging, with persistent barriers around governance, interoperability and data quality.

This Perspective paper draws on the experiences of the Developing research resources And minimum data set for Care Homes Adoption and use (DACHA) study which linked administrative health and social care records with records from care home software providers for over 700 older adult care home residents, an underserved population in research, in England to build a proof-of-concept minimum dataset.

From our learning, we make eight recommendations for researchers, research funders, data owners, data controllers and policymakers to strengthen future data linkage across health and social care. We recommend: (1) sharing metadata to support transparency and efficient reuse; (2) clarifying purposes for data sharing; (3) streamlining information governance processes; (4) recognising the health and social care system as a research partner; (5) resourcing data quality at the point of collection; (6) acknowledging the work needed to adapt routine data for research; (7) standardising core variables for interoperability; and (8) designing linkage for wider public benefit and safe data reuse.

Implementing these recommendations would help create a more coherent, efficient and equitable data landscape, realising the potential of existing data to improve care quality, research capacity and population health.

## Background

 Data linkage research using routinely collected data provides insights which cannot be realised in other research paradigms.^1^ As such, there is growing consensus and recognition of the necessity for improved data linkage across health and social care.^2 3^ The public are conditionally supportive of data linkage when this is secure and transparent.^4 5^ However, the general understanding of different data uses and the processes involved is low, and public engagement and involvement are recommended to establish and maintain support for linkage research.^4 5^

The challenges of undertaking large-scale routine data linkage research are well recognised by researchers, including the time required, overlap of processes and complexities in planning and resourcing.^3 6 7^ These issues are amplified where new data resources and cross-sectoral linkage are required.^8 9^ The response of the UK data research community to the COVID-19 pandemic showcased the potential benefits of large-scale data linkage, generating fast and strong evidence to inform care.^10^ This progress was achieved with temporarily expedited processes and governance based on emergency instructions to process confidential patient information—as detailed in the Health Service Control of Patient Information (COPI) Regulations 2002—which granted exceptional research powers for a limited time to address a global emergency.^11 12^

Globally, the maturity of population-wide data linkage for research varies significantly.^13^ In the UK, cross-sectoral linkages, for example, across primary care, secondary care, social care and community datasets, are currently limited to smaller geographies or population subsets.^13^,^15^ Herein, we will be reporting on a UK data linkage context across the breadth of health and social care (both private and publicly delivered), to ensure our recommendations are grounded in experience. Key terminology relevant to UK data sharing linkage is described in tables1  2.

**Table 1:** A quick guide to the different data roles under the UK GDPR

	Data controller	Data processor	Data owner*
Primary role	Decision-maker on why and how data are processed.	Executes processing tasks as instructed.	Ensures data quality, access control and compliance for a specific dataset.
Legal basis (eg, GDPR)	Defined and legally accountable under GDPR.	Defined and legally accountable under GDPR (to a lesser extent).	Not a GDPR role—used in data governance frameworks.
Accountability	Fully accountable for lawful data processing.	Accountable for following the controller’s instructions and ensuring security.	Accountable for internal data accuracy and integrity.
Decision-making power	Full control over data purposes and means.	No independent decision-making power.	Control over how data are managed and accessed within their domain.
Typical responsibilities	Define purpose of processing, ensure compliance, manage data subjects’ rights.	Store, analyse or transmit data as per contract.	Define data standards, ensure quality, manage permissions.
Example	A GP practice is the data controller for the patient data collected in their practice.	A cloud service stores hospital records.	The HR head manages employee records within their company.

* Data owner is not a GDPR role so these roles are not mutually exclusive.

GDPR, General Data Protection Regulation; GP, general practitioner; HR, human resources.

**Table 2:** Definitions of key terms relating to the use of data

Term	Definition (UK specific)	Key responsibilities/who is responsible
Routine health data	Health-related data that are collected regularly and systematically during the delivery of healthcare or administrative processes, rather than for research purposes. Examples include electronic health records (EHRs), hospital episode statistics (HES) and disease registries.	Data controllers (eg, National Health Service (NHS) organisations) ensure lawful use and secure storage; researchers must obtain appropriate approvals and justify use for secondary research.
Data protection impact assessment (DPIA)	A formal process to identify, assess and mitigate risks to individuals’ privacy when processing personal data, especially for new or high-risk processing. Required under UK GDPR and the Data Protection Act 2018.	Data controllers are responsible for conducting and documenting the DPIA; data protection officers (DPOs) provide advice and review outcomes.
Information governance (IG)	The system of policies, procedures and accountability structures that ensures information is handled legally, securely, efficiently and effectively within health and care organisations.	IG leads/Caldicott Guardians oversee IG compliance; all staff follow IG policies and complete training.
Data sharing agreement (DSA)	A legal document that sets out how, why and under what conditions data will be shared between organisations. It includes the purpose, legal basis, roles, security standards and retention periods.	Data controllers (of each organisation) jointly agree and sign; IG/legal teams draft and review; data processors must comply with terms.
UK General Data Protection Regulation (GDPR)	The UK’s version of the European Union (EU) GDPR, retained after Brexit and supplemented by the Data Protection Act 2018. It governs how personal data are collected, processed and protected within the UK, emphasising fairness, transparency, accountability and individuals’ rights.	Data controllers and processors must comply with data protection principles; DPOs ensure ongoing compliance and handle data protection queries.
Control of Patient Information Regulations 2002 (COPI Regulations)	UK regulations that allow the Secretary of State for Health and Social Care to require or permit the use of confidential patient information without consent for specific public health purposes (eg, disease control, emergencies or health service management). Often invoked during public health crises like COVID-19.	Health and care organisations must ensure data are used only within the scope of COPI notices; data controllers must document lawful basis and comply with security and confidentiality obligations.

Care homes in the UK provide 24-hour residential care and support for adults with complex needs, including services with and without on-site registered nursing staff. Data about their residents are separated across systems. Healthcare data are held on different systems by different parts of the National Health Service (NHS) while information about a resident’s stay in a care home is held by care homes (typically private companies) and by local authorities (financial information, needs assessments). With the drive to digitalise social care,^2^ there are a growing number of software providers providing solutions to care homes, creating further diversity of data sources.

People living in care homes are recognised to be underserved by data-driven research methods, typically due to difficulties identifying them as a complete population.^14^,^16^ Major efforts have been undertaken both in terms of algorithm development^17^ and more traditional research participant recruitment strategies,^10^ but neither offers a future-proof identification strategy using routine data sources. Despite significant data being collected, there are currently few links between different data sources that relate to care home residents,^15^ resulting in incomplete understanding of their needs, with siloed information often inaccessible to care providers. High-quality, accessible care home data are a priority for decision-makers at all levels, from individuals and their families through to commissioners and politicians. Such data could enable evidence-informed decisions for day-to-day service delivery and future planning for population change and crises such as pandemics.

In this paper, we will outline our experiences as researchers undertaking the Developing research resources and minimum data set for Care Homes Adoption and use (DACHA) study and wider linkage work,^18^ making eight recommendations to support accessing and linking data for research. Although DACHA relates to care home residents, these recommendations have broader relevance for researchers, research funders, data owners and policymakers across the health and social care sector (table 3).

**Table 3:** . List of recommendations

Recommendations	Target audience
1. Share metadata as a necessity for efficient and proportionate data reuse.	Data owners Data controllers Policymakers
2. Clarify purposes agreed for data sharing.	Data owners Policymakers
3. Streamline IG processes.	Data owners Data controllers Policymakers
4. Recognise the health and social care delivery system as a research partner.	Researchers Research funders Data owners Data controllers Policymakers
5. Provide resources to optimise primary data collection quality.	Data owners Data controllers Policymakers
6. Recognise the work required to adapt routine data for research use.	Researchers Research funders
7. Standardise the way core variables are recorded for interoperability.	Data owners Data controllers Policymakers
8. Design for wider impact of linkage on system and public benefit.	Data owners Policymakers

IG, information governance.

## The DACHA study

The DACHA study sought to create a proof-of-concept minimum dataset (MDS) for older peoples’ care homes in England.^18^ To minimise data burden and maximise use of existing data, we aimed to link resident-level care home-generated data from digital care records (DCRs) with a range of externally held data about those residents (including general practitioner (GP) records, community prescribing, social care, emergency care, secondary and community care data).^19^ A total of 45 care homes were recruited, with person-level informed consent to link 783 residents’ data (after exclusions), based on their NHS number (*individual-level identifier*), to undertake deidentified person-level and care home service-level analysis.^20^ The project was situated in three integrated care systems (ICS) at a time when these entities were newly established. ICSs are local health and care partnerships, established in July 2022, responsible for service planning and decision-making across distinct geographical areas.^21^ The project was ambitious as it sought to use a novel data source for research (DCRs from care homes using two distinct commercial software products); achieve cross-sectoral data linkage (joining social care and health data); and do so across three distinct geographies at a time of organisational change.

## Recommendations

Below we summarise the challenges we encountered and make eight recommendations to support future data linkages (table 3).

1. *Share metadata as a necessity for efficient and proportionate data reuse.*

By metadata, we mean an associated file, or data dictionary, which lists the variables recorded, their format (eg, integer, date) and any fixed or conditional responses (ie, variables only present based on earlier responses). There is significant variation in the availability of metadata for national datasets, which makes data mapping to fulfil UK General Data Protection Regulation (GDPR) principles and complete data protection impact assessments (DPIAs) difficult.

DCRs are commercial products which can be customised for care homes, for which there are currently no publicly available metadata. We overcame this in the study by limiting our study to care homes who used one of two software providers and working closely with them.

To facilitate the adaptation of routine data for novel research use, metadata should be recorded and made available for all those who could benefit from it. Inclusion of a brief guide to interpretation and limitations of these data will aid users. While private companies may not share their metadata publicly, availability of this on request would promote collaboration. Wider availability of metadata can facilitate more efficient and proportionate data reuse, minimising redundancy in data items and accelerating approvals by enabling specificity in requests.

2. *Clarify purposes agreed for data sharing.*

The collection and linkage of DCRs for DACHA, as a research study, was based on consent from individual residents or their consultees.^20^ This approach was needed within the constraints of a research project, but this would not be a scalable or proportionate approach to national routine collection of MDS data. We anticipated having individuals’ consent to link their data would enable data access, with appropriate data sharing agreements. However, consent did not help us to overcome existing restrictions on sharing of health records at national and ICS levels.

**Figure 1:**
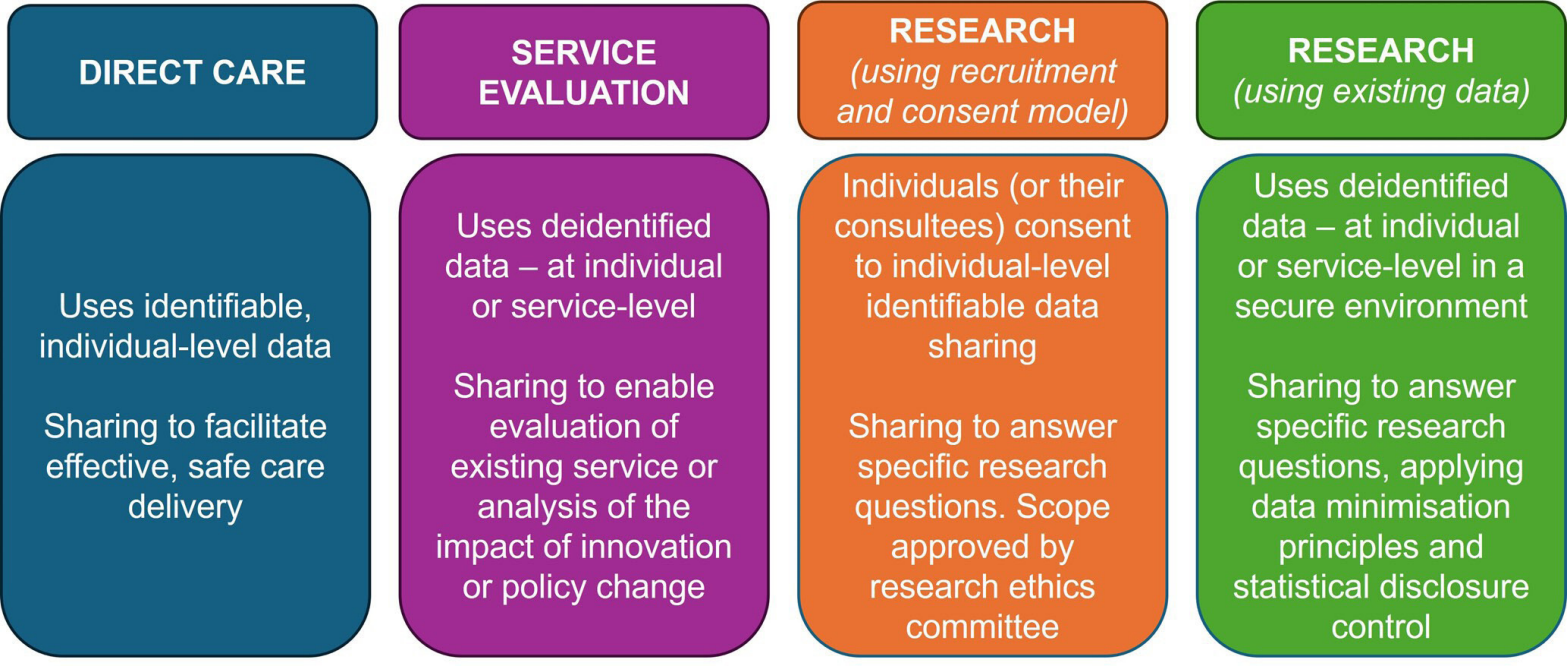
. Overview of data sharing purposes.

We worked with ICSs to access local, existing datasets, including GP data. Although some GP data were accessible to ICSs for population health management, there was no established route to gaining permission to access the data for research. The purposes for which the data could be shared (figure 1), along with the legal bases for data collection and sharing, were critical. A route was established in one ICS based on setting up data sharing agreements with individual GP practices, but ultimately, we were unable to link GP data within the time frame of the study.

ICSs are required to develop intelligence functions to support operational and strategic decision-making, underpinned by linked local data. Increasingly, cross-sectoral linkages are being established at a local level, but these linked datasets take significant time and effort to develop^22^ and are not necessarily set up for wider and secondary use or for linkage across different geographical areas.^23^,^25^

We recommend that new linked data resources should provide clear and public information about the purposes for which the data may be shared and the legal routes for safe reuse of data.

3. *Streamline information governance (IG) processes.*

DACHA stakeholders in NHS England (NHSE) and ICSs supported data linkage for our study in principle, with individuals generously sharing their time and expertise. However, delivering the technical aspects of data sharing required significant input from IG and business intelligence teams who had competing priorities for their time. At two of the ICSs, several different boards, groups and individuals were identified as needing to give approval. However, their approval was not sufficient to allow the work to be prioritised. Where we were able to access templates (such as for DPIAs) and learn about potential approaches to setting up data sharing, these were different in each ICS, so similar information had to be compiled in different ways.

For linkage of national-level NHSE datasets, we found that decision-making took time, and policies and processes were unclear. Gaining clarity required persistence, and we benefited from existing relationships with NHSE contacts. As changes made for one data controller required changes to all other data applications, uncertainty surrounding access to NHSE datasets meant that it was understandably difficult to persuade IG and analytical staff at ICSs to invest time discussing data access routes that may have to change.

Data controllers have good reason to be risk averse with respect to sharing person-level data in the interests of maintaining confidentiality, public trust and avoiding organisational harm and penalties. However, implementation of standardised application processes could simplify data sharing and reduce duplication, both for researchers seeking to access data and for data controllers, with no increased risk. Transparent processes would also allow research proposals to be planned and assessed fairly and equitably.

Secure data environments (SDEs) (also called trusted research environments) offer a potential solution to storage and security concerns while allowing safe and trusted collection and linkage of data, as well as helping to simplify access agreements and governance.^26^ In recent years, the NHS in England has committed to providing access to NHS data through an SDE Network, and there are now plans to create a new health data research service.^27 28^ Although these have the potential to streamline access to data, DCRs and records from other private providers of care are not included in SDE data collections.^22 25 26^ Since not all datasets may yet be linked within one SDE, streamlining of processes for existing, fragmented access routes remains important.

4. *Recognise the health and social care delivery system as a research partner.*

Although facilitating research is part of the ICS remit,^29^ resource pressures limited their capacity to support the DACHA study.^20^ Data processing and sharing, in addition to IG, require significant resource which ICSs were not able to prioritise against a backdrop of wider pressure.

A culture change to promote and resource partnership working across data controllers, data processors and researchers will place an emphasis on the reciprocal benefits of this relationship. A more collaborative approach should facilitate higher quality, relevant research.^30^ By including data controllers as copartners, resourcing costs can be accounted for within a funding application, and responsibility for research delivery shared. There has been significant progress formalising the necessary contribution of public participants in research through involvement and engagement. A similar approach could be used to integrate data controllers from the outset.

5. *Provide resources to optimise primary data collection quality.*

Improving the quality and usability of data at the point of collection is fundamental to strengthening the entire data pathway. Many of the datasets we accessed were of variable quality, particularly newer collections such as the Adult Social Care Client Level Data Set (ASC-CLD) and the national Ambulance Data Set. For example, we aimed to access data on falls, identified as a priority for the MDS, from the Ambulance Data Set. However, the field detailing the reason for an ambulance callout was 100% missing in our sample.^20^ This illustrates how weaknesses at the point of data capture can severely limit the usefulness of data for secondary analysis.

Effective data linkage also relies on the accuracy and completeness of core identifiers. In DACHA, we used deidentified NHS numbers and deidentified care home location as common identifiers. However, from a total of 783, we were unable to link records at person level for 16 residents and at care home level for 31 residents, due to missing or invalid identifiers.^20^ More broadly, many datasets that could enrich studies of life course and the wider determinants of health lack common identifiers preventing linkage. While probabilistic matching algorithms using multiple identifiers can sometimes compensate, this approach still relies heavily on the completeness, consistency and validity of the underlying data.

Improving data quality must therefore begin at the point of primary data collection with targeted investment in infrastructure, technology and workforce capacity. Systems that support consistent data entry—through well-designed digital tools, data standards and validation prompts—should be coupled with adequate time, training and support for staff responsible for recording data. However, sustained improvement relies on ensuring that data collection has tangible value for those entering the data. When local decision-makers and frontline staff can use the information to guide service delivery, planning and improvement, there is stronger motivation to record data accurately and completely.^31^

6. *Recognise the work required to adapt routine data for research use.*

In DACHA, we worked directly with two software providers to understand what information was collected in DCRs, and what could be extracted for research use.^20^ The software allows staff to complete both structured data and free text. The latter could not be shared due to the potential inclusion of identifiable information but may have held information, such as ethnicity or dementia status, that was inconsistently recorded in structured fields.

While routine data sources, such as DCRs, offer an efficient data source for research,^32 33^ turning them into usable research resources requires additional effort, often not appreciated. Understanding the context of data collection—who recorded the data, for what purpose and under which operational pressures and data standards—is essential as these data are primarily generated to support care, not research.^32 33^

High-quality research use requires dedicated work to characterise and validate datasets.^34^ This includes exploring how variables are defined and used in practice, identifying potential biases and assessing completeness and accuracy. Even when metadata exist, additional investigation is needed to understand the meaning and reliability of fields.

Funders and researchers should therefore set realistic expectations and allocate specific resources for this foundational work. When using new or evolving data sources, the effort required to make them research ready can be substantial. Investment in data curation, documentation and quality improvement enhances the validity of findings and builds a shared, reusable resource that benefits multiple research projects and reduces duplication of effort (eg, public codelists, GitHub scripts).

7. *Standardise the way core variables are recorded for interoperability*.

In DACHA, data extracted from DCRs included variables anticipated to be routinely collected, such as weight and height, as well as measures added and recorded for the study (eg, quality of life), using detailed specifications. However, the two DCR systems differed in how they handled variables (including those added for DACHA): variables were often coded differently across the two systems, or collected by only one; and repeat inputs were either presented as a blank field for completion or were prepopulated with the most recent information. As a result, the cleaning and standardising of DCRs was more time consuming than expected, despite close working with software providers to specify formats.^20 33^

There are over 50 software providers providing software solutions to care homes. Currently, they offer systems bespoke to the care home. Although helpful to care homes, this has the potential to impede the ability to gain system-wide insights. The Department of Health and Social Care (DHSC) has sought to address this, to some extent, by introducing a Minimum Operational Data Standard (MODS) in April 2024.^35^ The MODS will require consistent recording of a set of core variables across all Care Quality Commission-registered adult social care providers in England according to a standardised technical specification, similar to the approach seen for independent sector providers in healthcare who input into hospital data systems.

Standards for digital health and social care records, for example, those developed by organisations such as the Professional Record Standards Body and the MODS,^35^ will help facilitate interoperability. We consider that standardisation of at least a core set of variables in each domain of health and care is important to enable learning,^36^ even if customisation to reflect local needs and practice can still be valued and beneficial. Ideally, standards would be developed before widespread rollout, to minimise administrative burden, and services and software providers would be supported to implement changes alongside enforcement by DHSC.

8. *Design for wider impact of linkage for system and public benefit*.

Inclusion of GP data in the DACHA MDS was important, but not achieved within the time frame of the study. Access was particularly challenging because of the need to gain approval from individual GP practices. Although data from large numbers of GP practices can be accessed and linked to selected datasets via Clinical Practice Research Datalink (CPRD), OpenSafely and others, at present there is no established route to link GP data with bespoke external data sources such as DCRs, although this is a rapidly changing area.^10 37 38^

Linkage of DCRs with other health datasets was also challenging, as we found that linkages already set up at ICS level could not easily be repurposed for external research.^22 24^ DHSC has launched the national ASC-CLD, a subset of data on local authority-funded social care held by NHS England, as a first step towards a long-term goal of improved cross-sectoral linkage, and there are plans to include social care data from local authorities in NHS England’s SDE Network.^28^ However, there are no plans to link DCRs with a wider range of datasets in a routine way.^2^ Linked administrative datasets have been established in ICSs, for example, in Greater Manchester and Salford, and North East London. However, it is not currently possible to link these datasets with DCRs or across geographical areas. In addition, linked data are often required to be deleted after completion of research, with no provision for reuse, missing opportunities to capitalise on the work to establish novel linkage.

Public benefit should be the organising principle of study designs and development of new datasets or linkages. We recommend that policymakers and data owners introduce appropriate legal routes and safeguards for the safe reuse of data, for a range of purposes including commission/planning, service improvement and research, similar to legal frameworks that already exist for routinely collected data in the NHS, and prioritise interoperability in data collection, to maximise the future potential of the data.

## Conclusions

Reflecting on our experience undertaking novel, cross-sectoral linkage across geographies in England, we have made eight recommendations to support accessing and linking data for research in the UK. While some echo others who have traversed this complex landscape before us, reflecting on time, resources and complexity, we also highlight the need for transparency, openness, consistency, partnership working and planning to ensure greater public benefit from research endeavours.

The UK is rich in routine data, and the potential to harness this existing information to achieve public benefit is vast. However, just because data exist does not mean that repurposing is simple or rapid to achieve. Recognising this and planning accordingly is necessary to realise the goal of linking a broader range of datasets to better understand the needs, experiences and outcomes of the population living in care homes and beyond. Implementing these recommendations could realise the potential value of existing data in a more equitable, cost-effective and timely way, allowing those funding, undertaking and facilitating research to derive more insights to improve care.
